# Diagnostic Imaging Characteristics of a Rare Primary Ovarian Mesonephric-Like Adenocarcinoma: A Case Report and Literature Review

**DOI:** 10.7759/cureus.79780

**Published:** 2025-02-27

**Authors:** Nanami Suzuki, Kenro Chikazawa, Fumi Kato, Shiori Ando, Naota Okabe, Ken Imai, Tomoyuki Kuwata

**Affiliations:** 1 Department of Obstetrics and Gynecology, Jichi Medical University, Saitama Medical Center, Saitama, JPN; 2 Department of Radiology, Jichi Medical University, Saitama Medical Center, Saitama, JPN; 3 Department of Diagnostic Pathology, Jichi Medical University, Saitama Medical Center, Saitama, JPN

**Keywords:** adenocarcinoma, magnetic resonance imaging, mesonephric-like adenocarcinoma, ovary, preoperative diagnosi

## Abstract

Ovarian mesonephric-like adenocarcinoma is rare and poorly understood, and preoperative diagnosis of this tumor with any diagnostic modality is challenging. Histological features can only be speculated through imaging. Herein, we present the case of a 64-year-old woman with primary ovarian mesonephric-like adenocarcinoma, characterized by a 13 cm pelvic mass detected via ultrasound. Further evaluation with magnetic resonance imaging showed low signal intensity in the solid component on T2-weighted images and high signal intensity on diffusion-weighted images, along with a smooth margin; these findings are consistent with those of previous reports. The patient underwent a total abdominal hysterectomy, bilateral salpingo-oophorectomy, and partial omentectomy. Postoperative pathology confirmed mesonephric-like adenocarcinoma with positive peritoneal cytology. Immunohistochemical analysis showed a positive result for GATA-3 and a negative result for thyroid transcription factor-1. The patient was diagnosed with stage IC3 mesonephric-like adenocarcinoma, and adjuvant chemotherapy with paclitaxel and carboplatin was initiated. Mesonephric-like adenocarcinoma can present with distinct imaging features, including low signal intensity on T2-weighted imaging and high signal intensity on diffusion-weighted imaging, with a smooth solid margin; these imaging features may aid in the preoperative differentiation from other ovarian malignancies, such as serous or clear cell carcinomas.

## Introduction

Mesonephric-like adenocarcinoma (MLA) has been classified as a primary ovarian carcinoma according to the World Health Organization's 2020 classification [1,2]. It is primarily diagnosed postoperatively, with limited reports on its preoperative imaging findings and no cases been definitively diagnosed based on preoperative imaging [3-5]. There is a lack of comprehensive reports detailing preoperative imaging characteristics of MLA. However, a review of existing literature revealed some shared imaging characteristics among reported cases, which may aid in recognizing MLA as a potential differential diagnosis. Herein, we present the case of primary ovarian mesonephric-like adenocarcinoma and review previous reports on imaging-based diagnoses [3,6-8].

The study was approved by the Ethics Review Board of our institution (S24-085), and informed consent was obtained from the patient for the publication of this report and associated images.

## Case presentation

**Figure 1:**
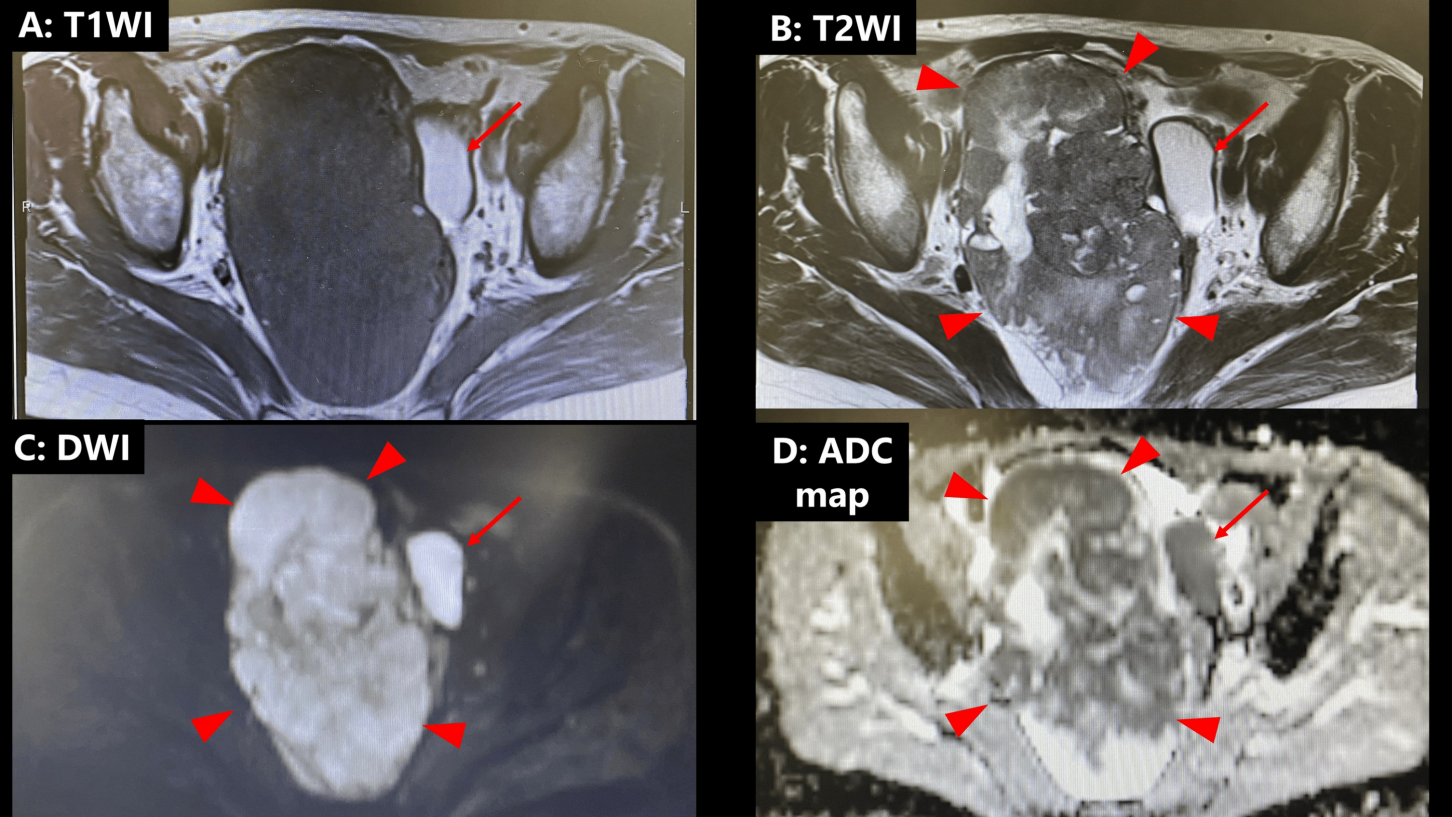
Magnetic resonance imaging features The solid components of the right ovarian tumor show significantly low signal intensity on T2-weighted images (B). Diffusion-weighted images show high signal intensity in the solid region (C). The solid part exhibits a smooth margin. The solid components show low ADC values (0.535×10-3 mm2/s) on the ADC map (arrowhead) (D). The left ovarian cyst shows high signal intensity on both (A) T1- and (B) T2-weighted images and suggests a hemorrhagic ovarian cyst (arrow). Due to the high signal intensity on DWI and low ADC values, along with the presence of a cystic lesion, the diffusion restriction likely reflects hemorrhage (C, D) [9]. T2WI, T2-weighted imaging; DWI, diffusion-weighted imaging; ADC, apparent diffusion coefficient

A 64-year-old woman (gravida 3, para 2) with no relevant medical or family history presented with abdominal distension to our institution in March 2025. A 13 cm pelvic mass was detected on transvaginal ultrasonography. Further evaluation with magnetic resonance imaging (MRI) revealed a mass measuring 133 × 121 × 80 mm in the right ovary. The right ovarian tumor exhibited heterogeneous signal intensity on T1-weighted images, rather than uniform signal intensity typically observed in fibroma. On T2-weighted images (T2WI), the solid components demonstrated significantly low signal intensity, while other areas showed variable signal intensities with a small cystic component (Figure 1).

Diffusion-weighted images (DWI) showed high signal intensity in the solid regions. The apparent diffusion coefficient (ADC) value of the solid components was 0.535 × 10-3 mm²/s. The solid part exhibited a smooth margin. Given these findings, ovarian cancer was suspected, and the patient was referred to our department for surgery.

Whole-body computed tomography revealed no obvious metastases or lymphadenopathy. Tumor markers were elevated, with cancer antigen 19-9 at 287.5 U/mL and cancer antigen 125 at 206.3 U/mL, but other blood test results were unremarkable.

The patient underwent a total abdominal hysterectomy, bilateral salpingo-oophorectomy, and partial omentectomy for suspected ovarian cancer. Intraoperative frozen section analysis suggested adenocarcinoma with uncertain histology. No peritoneal metastases were observed; however, capsular rupture occurred intraoperatively. Pelvic and para-aortic lymph node dissection was not performed at the patient’s request.

**Figure 2:**
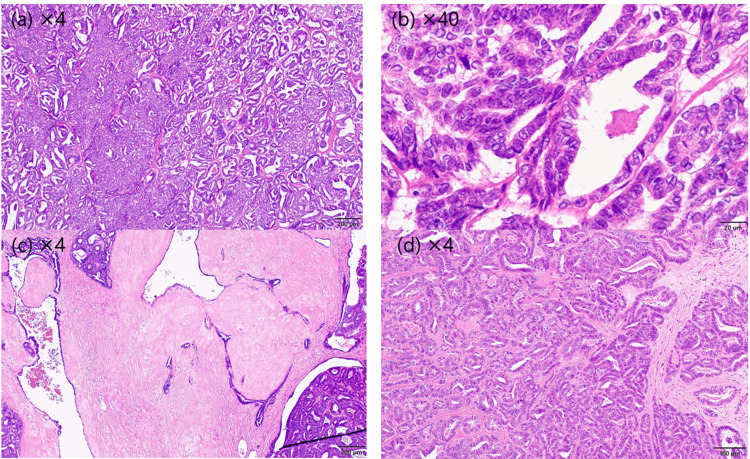
Histological features Histological findings reveal tumor proliferation with tubular, ductal, and papillary patterns (a,b). The tumor glands are filled with eosinophilic colloid-like material (b). Abundant fibrous stroma is recognized between the tumor glands (c,d).

**Figure 3:**
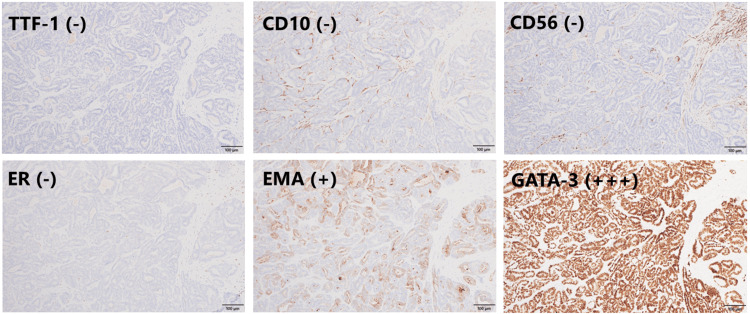
Histological features Immunohistochemically, tumor cells are positive for GATA-3 and EMA, while they are negative for ER, TTF-1, CD10, and CD56.

Postoperative pathological examination confirmed a diagnosis of MLA (Figures 2, 3).

Peritoneal washing cytology was positive. Hematoxylin and eosin staining revealed histological tubular structures containing eosinophilic material. Immunohistochemically, most tumor cells were positive for GATA-3 [4]. Although thyroid transcription factor-1 is often positive in MLA [7], it was negative in the present case. CD10, CD56, and ER were also negative, while epithelial membrane antigen was positive.

The final pathological diagnosis was stage IC3 ovarian MLA. The patient had been scheduled for six cycles of adjuvant chemotherapy with paclitaxel and carboplatin.

## Discussion

In the present case, MRI revealed that the solid component exhibited low signal intensity on T2WI and high signal intensity on DWI, and a smooth margin. These imaging features are consistent with previously reported findings, suggesting their potential utility in preoperative diagnosis of MLA [6,7]. These imaging findings are summarized in Table 1, which consolidates the reported characteristics of MLA on MRI, highlighting the consistent presence of low T2WI signal intensity, high DWI signal intensity, and a smooth solid component, which may assist in distinguishing MLA from other ovarian malignancies. While these imaging features have been consistently reported, we acknowledge that they are not entirely specific to MLA and may overlap with the characteristics of other ovarian tumors. Further accumulation of cases and comparative studies are needed to validate their diagnostic utility.

**Table 1:** Magnetic resonance imaging characteristics of mesonephric-like adenocarcinoma T2WI, T2-weighted imaging; DWI, diffusion-weighted imaging

Study	T2WI	DWI	Morphology of the solid part
Lee et al. 2024 [7]	Low	High	Smooth
Yang et al. 2024 [3]	Low	High	Smooth
Ujita et al. 2021 [6]	Low	High	Smooth
Present case	Low	High	Smooth

To further understand the imaging characteristics of MLA, we conducted a literature review on October 1, 2024 using PubMed, with “mesonephric-like adenocarcinoma” and “ovary” as the keywords. No date restrictions were applied, and all available articles published up to the search date were included. This search yielded 50 articles; among them, only three provided actual imaging data. Based on the imaging findings from these three articles, we have included the literature review as a result section before the discussion section to enhance clarity. Additionally, we acknowledge the limited availability of imaging-focused studies on this topic (Table 1). Notably, our case presented characteristic imaging features similar to those in previously reported cases, including low T2WI signal intensity, high DWI signal intensity, and a smooth solid component. However, in previously reported cases in our literature review, MLA was postoperatively diagnosed with histopathological examination, with no cases being identified based on preoperative imaging findings. A common feature between the present and previously reported cases was the presence of fibrous stroma between glandular structures, which showed significantly low signal intensity on T2WI. Morphologically, the solid tumor component appeared smooth, without irregular papillary projections [6,7]. Additionally, the high signal intensity on DWI observed in our case aligns with findings in previously reported cases.

However, these imaging features are not entirely specific to MLA, as similar findings may be observed in other ovarian tumors, such as struma ovarii and endometrioid adenocarcinoma. In particular, the stained-glass appearance, while noted in MLA, has also been described in struma ovarii and mucinous adenocarcinomas. Although MLA shares some imaging characteristics with other ovarian malignancies, it also presents distinct differences. For example, clear cell carcinoma often exhibits high signal intensity on T2WI due to its glycogen-rich cytoplasm, whereas MLA typically demonstrates low signal intensity on T2WI due to its fibrous stroma. Similarly, endometrioid carcinoma frequently displays irregular solid components with papillary projections, unlike the well-defined, smooth solid component seen in MLA. The presence of a well-defined, smooth solid component and significantly low signal intensity on T2WI may aid in differentiating MLA from these entities. Nonetheless, further case accumulation and comparative analysis with other ovarian neoplasms are necessary to better establish the diagnostic utility of these imaging characteristics. In conventional imaging, low signal intensity on T2WI and high signal intensity on DWI often suggest a metastatic tumor. However, the thecoma-fibroma group does not display high signal intensity on DWI, which may aid in differentiation [8]. These distinguishing features are summarized in Table 1.

While positive peritoneal cytology and elevated tumor markers are commonly associated with aggressive or advanced-stage ovarian malignancies, their clinical significance in MLA remains unclear due to the rarity of this tumor. Given the limited number of reported cases, currently, no established correlation exists between MLA and peritoneal cytology status or specific tumor markers. Further accumulation of cases is required to determine whether these factors have prognostic or diagnostic value in MLA.

## Conclusions

While imaging reports on MLA are limited, notable features, such as low signal intensity on T2WI, high signal intensity on DWI, and a smooth solid component, have been observed. These findings suggest that MLA exhibits a distinct imaging pattern that may aid in preoperative diagnosis. However, larger case studies are needed to confirm their diagnostic reliability.
